# Supplementary material for: Perturbation biology links temporal protein changes to drug responses in a melanoma cell line
Source: PLoS Comput Biol. 2020 Jul 15;16(7):e1007909. doi: 10.1371/journal.pcbi.1007909 (PMC7384681; doi:10.1371/journal.pcbi.1007909)
Supplement: Attachment — Submitted filename: Response_to_Reviewers_PLoSCB_resub_1st_round.pdf [file pcbi.1007909.s014.pdf]

DANA-FARBER
CANCER INSTITUTE

Response to reviewers:

Reviewer #1

This is a well executed study covers a good mix of both computational and biological aspects of perturbation biology and is thus a good fit for PLoS Comp Bio. The authors introce an RPPA dataset covering the time resolved response of 126 markers to 54 drug combinations and train, validate and test a semi-mechanistic, datadriven ODE-type model on this dataset. They illustrate how the model rederives known drug-target interactions and introduce a measure of importance for individual nodes. The then go on to validate the importance of individual nodes and node combinations experimentally. The paper is well written and easy to follow, altough the some aspects of the study are not covered in sufficient detail, which I outlined below. Besides that I have noted a couple minor technical aspects that I think should be adressed.

Response*: We thank the reviewer for the positive evaluation of our manuscript. We have addressed specific questions below and also improved the general quality of manuscript.*

MAJOR COMMENTS:

1. Why did the authors decide to evaluate the model on the test set based on correlation in contrast to RSS in the previous analysis? I think looking at correlation is certainly valuable, but seems a bit inconsistent with the previous analysis. Both correlation and RSS have its pros and cons so analysing both would be valueable. Moreover it would be interesting to see the dependence of model predictions on drug/marker to complement the timepoint analysis.

Response: *We agree that adding RSS is informative and have included it side by side with the previously reported Pearson correlation coefficient. The corresponding mean RSS value for all data is 0.181 (compared to the Pearson's correlation coefficient of 0.54) and 0.118 for phenotype only (compared to 0.79). This information has also been added to Figure 3 and the main text. We also agree that it is valuable to repeat the procedure of model selection and validation using an alternative metric and have therefore added a corresponding Pearson correlation analysis for the validation data set (see new Figure S4).*

2. How much is RSS biased based on these strong outliers with values < -4? Overall I am bit surprised by the number of datapoints with values smaller than -3 given that the number such datapoints in figure 2 seems to be very small.

Response: *Given the fact that we use a fixed split of training, test and validation set, even models with an increased complexity relative to the ones selected will not be able to predict the outliers in the test data set correctly (Figure 3), which would reduce the value of the RSS, i.e., the model selection is not affected by these. We have verified the data and can confirm that the displayed points with values below −3 are contained in the displayed data of Figure 2. As an*

Richard Stein, Ph.D. • Research Associate HSPH • Dana-Farber Cancer Institute • Biostatistics and Computational Biology
450 Brookline Avenue • Boston, MA 02215-5450 • Tel: 617-582-7229 • stein@jimmy.harvard.edu

example, the drug combination of RAF and PKC inhibitors shows few "outliers" in early time points. In general, the drug combinations with a low response have strong outliers (as we normalize to control measurements).

3. How do the model predictions compare to simple null models for drug interaction such as bliss independence or highest single agent?

Response: *We have looked for drug synergies in our data set using the specified methods. Since we did not identify significant and interesting synergies, we have not included this analysis in the manuscript. Instead, we have focused on our modeling approach to predict the effect of novel target inhibitions. For most of these predictions, we do not have the corresponding data (with the exception of the validation in Figure 6). The question, in our interpretation, can therefore not really be answered without further experimental efforts, i.e., we cannot compute synergies for the new combinations of target inhibitions using the simple null models without including more data.*

4. Regarding the optimization method I was wondering what the motivation was to crop interaction parameters during the optimization process. Using a gradient based method, this may lead to poor optimizer performance as it introduces discontinuities in the objective function. Why not just crop values after optimization is done? Its also unclear how derivatives are computed for cropped parameters?

Response: *The use of parameter cropping during the optimization has the advantage of lowering computational runtime as well as finding reliable and sparse solutions as a function of the regularization parameter lambda. We are aware that cropping implies the necessity to select a "cropping threshold", and different thresholds may lead to different selected parameter sets. However, we found that the cropping during optimization was well behaved, despite the discontinuities, and we therefore believe the stochastic gradient descent method is nevertheless able to solve the gradients. We have reworked and extended the corresponding paragraph in the manuscript's method section (lines 408–413). Although it would be interesting to do a systematic analysis of cropping during and after optimization using different thresholds, we think such an analysis is beyond the scope of the current paper.*

5. I am not entirely sure what the authors are doing for the analysis presented in figure 5. The methods section is a bit vague on what was actually done. Were curves to compute EC50 values fit to simulation results? How were individual EC50 values extrapolated to combinations handled? I believe both methods description and results section need to be a bit more verbose about methods and rationale.

Response: *We agree that the method description requires more detail and have expanded the description in the text (line 220–227). In summary, we simulated all individual network models with different levels of input strength of an in silico inhibitor for each target present in the model. We focused on the effect on cell growth and apoptosis for these input strengths and calculated*

Richard Stein, Ph.D. • Research Associate HSPH • Dana-Farber Cancer Institute • Biostatistics and Computational Biology
450 Brookline Avenue • Boston, MA 02215-5450 • Tel: 617-582-7229 • stein@jimmy.harvard.edu

DANA-FARBER
CANCER INSTITUTE

their mean values (presented in Figures S6 and S7). From these curves, we further calculated the dose that gave rise to the half maximal response (EC_{50}). This EC_{50}-dose was used, both alone for each target and in all combinations of pairwise targets, to simulate the predictions in Figure 5. To be clear on the reviewers comment: we did not combine individual EC_{50} responses to get the combined response – we combined the doses that were calculated from the dose response simulations and performed new simulations for all of those combinations.

6. Figure 6 needs better labels or colorcoding, it is very difficult to track what is what. Its unclear how observed maximal response values were computed (bottom/right). Is this log2 fold change on the cell growth node? Shouldnt normalized cell count be the result of cell growth and cell death? The methods section mentions something about "equivalencing", but I am not sure what this is supposed to mean and whether this is relevant to this figure.

Response: *We have now improved the colorcoding and labelling of Figure 6. With cell growth, we mean the normalized cell count, i.e., the number of cells under drug treatment divided by the number of cells in the unperturbed control. Another definition of growth could be related to death and/or proliferation. In addition to the number of cells we measure a marker of apoptosis experimentally in two independent measurements. Indeed, we are in log_2-space for both cases of reporting all data and all of the model predictions. We have now clarified this for both Figures 5 and 6.*

7. Although I don't want to question it the equivalence of drugs with their target nodes in the model is nontrivial and deserves a bit more explanation/justification.

Response: *Indeed, drugs and their relation to the specified targets is a complex issue, and we agree that our previous description was lacking thorough discussion of this topic. We have added a more detailed discussion in the revised version and added a paragraph to the Results section, in which we relate our model-based drug predictions to literature data (lines 278–289).*

8. The level of technical documentation about the employed ode solver seems a bit shallow for a computational journal. It may be adequate to cite the recent preprint on Interpretable Machine Learning for Perturbation Biology by the authors as more detailed reference for the employed methodology. The tensorflow optimization seems to use an explicit euler scheme without stepsize control for integration, which seems a bit unorthodox given the fact that many biological systems exhibit timescale separations which renders the underlying equations stiff. The model formulation and parameter boundaries may prevent this from happening, but it would be reassuring to validate the correctness of solutions with a state of the art ode solver with implicit integration scheme and adaptive step-size control such as scipy.integrate.solve_ivp (python) or ode15s (matlab)

Response: *We agree that the article would benefit from more detail about the modeling procedure and have now included these details into the Method section (lines 403/404 and 439–443). In summary, we used a two-stage Runge–Kutta method with a fixed step size, since none*

Richard Stein, Ph.D. • Research Associate HSPH • Dana-Farber Cancer Institute • Biostatistics and Computational Biology
450 Brookline Avenue • Boston, MA 02215-5450 • Tel: 617-582-7229 • stein@jimmy.harvard.edu

of the state of the art solvers were present in Tensorflow at the time of implementing the optimization. We have compared the obtained networks to those obtained when using ode15s in Matlab and obtain similar network models.

9. In the discussion the authors claim. "Therefore, to avoid mis-interpretation of predictions, it is important to always study a set of obtained network models, and not only the single best solution". Although I agree with this notion, the authors don't seem to follow their own advice to closely, at least according to what was described in the paper. I would be good to know at which point uncertainty of predictions was evaluated in this study and when model averaging etc was performed. The only figure in which I could spot errorbars for multiple model realizations was in figure 3. The authors should also note that just running multiple optimization runs does not warrant proper uncertainty analysis (c.f. "Uncertainty Analysis for Non-identifiable Dynamical Systems: Profile Likelihoods, Bootstrapping and More"). I am aware that proper uncertainty analysis using profile likelihood or bayesian methods is not realistic for models of the considered size and the authors approach is thus justified, but I think the paper should explicitely state this in the discussion.

Response: *We thank the reviewer for raising this discussion point and have followed the suggestion to mention these points in the Discussion section and have also included the suggested reference (lines 356–362). Many of the presented results are based on averages for practical and visual reasons. When the visualization style allowed, errorbars were included. However certain visualizations (for example heatmaps) preclude an easy indicator of uncertainty. If possible, we have considered the observed (but of course not the total) uncertainty in our analysis, for instance, in Figure 3 as pointed out by the reviewer, as well as in Figures 6, S4, S10, and S11, which also include the observed model uncertainty.*

MINOR COMMENTS:

10. Some supplemental figures are blurry in preview, I believe thats a matlab artefact with known workaround?

Response: *Thank you for pointing this issue out. This problem has now been fixed for Figures S1 and S2.*

11. The importance of EGFR in the model highlights the importance of paracrine signaling in drug response. Accordingly, the authors may want to reconsider treating cell death/growth as terminal nodes in the model given that both may affect the degree of paracrine signaling. I don't think this needs to be adressed within the scope of this paper though.

There are still a couple of typos in the manuscript, the authors may want to recheck the manuscript.

Richard Stein, Ph.D. • Research Associate HSPH • Dana-Farber Cancer Institute • Biostatistics and Computational Biology
450 Brookline Avenue • Boston, MA 02215-5450 • Tel: 617-582-7229 • stein@jimmy.harvard.edu

DANA-FARBER
CANCER INSTITUTE

Response: *We thank the reviewer for raising these points and agree that it is out of scope. Regarding the latter point, we have significantly improved the manuscript in the revision process and have corrected the typos.*

Reviewer #2

In this manuscript titled Perturbation biology links temporal protein changes to drug responses in a melanoma cell line, Nyman et al. presented a completely data-driven approach of drug response prediction, building upon their previously developed framework of modeling the dynamic changes of cellular molecules under perturbations with a series of coupled nonlinear ordinary differential equations. They fitted the model using time-dependent RPPA data under various drug (combination) treatments in the A2058 melanoma cell line, applied the model to propose efficient novel drug treatments, and experimentally validated the proposed treatments. Overall, we find the work quite novel and interesting.

We have previously reviewed this manuscript during the authors' submission to another journal. We are glad to see and appreciate that the authors have properly corrected some of the issues we brought up last time and largely improved the manuscript. However, we feel that some of our previous concerns have not been sufficiently addressed and therefore suggest a further revision. Here we re-discuss these issues as follows.

First, to recapitulate our previous major comments:

The authors made predictions on drug effects based on both cell growth and apoptosis, however it seems that the authors selected and tested particular drugs (and drug combinations) only based on the effect on cell growth ("normalized cell count" as in Figure 6). For completeness it is desirable to have additional validations specifically of the predicted drug effects on apoptosis (with caspase fluorescent assay). This can also be important since some perturbations (e.g. EGFR inhibition, Figure 5) were predicted to strongly suppress cell growth but only weakly trigger apoptosis. If validated it may testify the additional value of the model in revealing the context-specific mechanism of the drug action.

Most of the predictions the authors chose for validation are positive cases, i.e. where the treatment is predicted to be effective. While this can provide measures of sensitivity of the predictions, the specificity of the predictions is not well-accessed. It can be desired to test a few more cases of negative prediction to evaluate the false positive rate.

Essentially, our consideration underlying both of the above two comments is that experimentally testing the "negative cases", although not interesting for application, is nonetheless important in thoroughly evaluating the method. Based on the limited one or two negative cases the authors have already tested, we have a concern that the method may suffer from low specificity. Moreover, testing the negative cases are not entirely biologically meaningless, e.g. in comment

Richard Stein, Ph.D. • Research Associate HSPH • Dana-Farber Cancer Institute • Biostatistics and Computational Biology
450 Brookline Avenue • Boston, MA 02215-5450 • Tel: 617-582-7229 • stein@jimmy.harvard.edu

DANA-FARBER
CANCER INSTITUTE

1, if neutral effect on apoptosis can be validated, it will provide mechanistic insight for the action of the drug (since it does inhibit cell growth), and will help to demonstrate extra values of the authors' method. In summary, we therefore think that it's desirable to address at least one of the above comments. If additional biological experiments are not feasible, we think the authors may at least try to validate some of these by comparing to published data or literature and provide a proper discussion of these issues.

Response: *We thank the reviewer for the positive feedback in the current and previous review and agree that more experimental validation would be essential to greatly advance any work in this direction – although out of scope for this manuscript. As a way to resolve this, we now added a subsection "Comparison with literature data" focusing on literature-based discussion of the predicted drug effects to the Results section (lines 278–289). Moreover, we included a comparison of data vs. drug sensitivities (additional supplementary tables S2 and S3 with corresponding Figure S10) based on data from the Wellcome Sanger Institute for the cell line used in our study (A2058). The new analysis provides further insight into the complex task of predicting the outcome of target inhibition. As highlighted here, different drugs with the same target may have opposite effects on the cell growth. Using this additional data set, our predictions are confirmed such that we do not find obvious false positive model predictions, i.e., cases in which we have predicted a protein target to be efficacious, while the data shows the opposite. Regarding the suggestion to address predicted apoptosis responses with corresponding measurements: those data are harder to find in the published literature, but we agree that it would be of great value to design an experimental study in which we compare our model predictions of apoptosis with corresponding experimental data. In addition to the points above, we extended the Discussion section to explain more of the assumptions and possible limitations of our model predictions (lines 335–353).*

Richard Stein, Ph.D. • Research Associate HSPH • Dana-Farber Cancer Institute • Biostatistics and Computational Biology
450 Brookline Avenue • Boston, MA 02215-5450 • Tel: 617-582-7229 • stein@jimmy.harvard.edu
